# Resveratrol and Redox Regulation in Cardiovascular Disease Across the Life Course: Mechanistic and Translational Perspectives

**DOI:** 10.3390/antiox15040509

**Published:** 2026-04-20

**Authors:** Chien-Ning Hsu, You-Lin Tain

**Affiliations:** 1 Department of Pharmacy, Kaohsiung Municipal Ta-Tung Hospital, Kaohsiung 801, Taiwan; cnhsu@cgmh.org.tw; 2 Department of Pharmacy, Kaohsiung Chang Gung Memorial Hospital, Kaohsiung 833, Taiwan; 3 School of Pharmacy, Kaohsiung Medical University, Kaohsiung 807, Taiwan; 4 Department of Pediatrics, Kaohsiung Chang Gung Memorial Hospital, Kaohsiung 833, Taiwan; 5 College of Medicine, Chang Gung University, Taoyuan 333, Taiwan; 6 Doctoral Program of Clinical and Experimental Medicine, National Sun Yat-Sen University, Kaohsiung 804, Taiwan

**Keywords:** cardiovascular disease, resveratrol, developmental origins of health and disease (DOHaD), drug delivery, oxidative stress, polyphenol, cardiovascular–kidney–metabolic syndrome

## Abstract

Resveratrol (RSV), a bioactive polyphenol, has emerged as a pleiotropic modulator within the integrated pathophysiology of cardiovascular disease (CVD) across the life course. Effective CVD management requires a transition from organ-centric frameworks to systems-level models that acknowledge dynamic crosstalk among metabolic, renal, and cardiovascular networks. Oxidative stress constitutes a central unifying axis in this interconnected biology, propagating cross-organ injury from early developmental stages onward. Mechanistically, RSV acts as a redox-responsive gene regulator by activating the Nrf2–ARE pathway, restoring nitric oxide bioavailability, and orchestrating SIRT1, AMPK, and NF-κB signaling to recalibrate mitochondrial function, inflammatory tone, and endothelial integrity. Within the Developmental Origins of Health and Disease (DOHaD) paradigm, RSV exhibits reprogramming potential that attenuates the intergenerational transmission of hypertension, kidney disease, and metabolic dysfunction. Although clinical translation is constrained by limited bioavailability and rapid metabolism, advanced delivery systems and artificial intelligence-enabled optimization strategies provide promising avenues to enhance therapeutic precision and scalability. This narrative review integrates mechanistic and translational insights to position RSV as a systems-oriented life-course intervention with sustained and intergenerational relevance in CVD.

## 1. Introduction

Resveratrol (RSV), a natural polyphenolic compound abundant in grapes, berries, and peanuts [1], has attracted considerable interest as a pleiotropic modulator of cardiovascular health [2,3]. However, effective life-course management of cardiovascular disease (CVD) requires a paradigm shift from organ-specific treatment to an integrated framework that captures the dynamic interplay between metabolic, renal, and cardiovascular dysfunction across the lifespan. In this context, the concept of cardiovascular–kidney–metabolic syndrome (CKMS) has emerged to capture the integrated pathobiology linking obesity, diabetes, chronic kidney disease (CKD), and CVD, thereby better reflecting real-world disease trajectories [4,5]. Rather than viewing CVD as an isolated end-stage event, CKMS conceptualizes cardiovascular risk as the cumulative consequence of interconnected metabolic and renal disturbances that begin early and progress over time.

These conditions share convergent risk factors and interconnected mechanisms, including oxidative stress [6], chronic inflammation [7], nitric oxide (NO) dysregulation [8], and gut microbiota imbalance [9,10], which collectively drive disease clustering and accelerate adverse outcomes. Among these mechanisms, oxidative stress functions as a central unifying axis within CKMS, amplifying cross-organ injury and perpetuating cardiometabolic–renal crosstalk. Increasing evidence that oxidative stress represents a pivotal pathway in CKMS progression has renewed interest in antioxidant strategies with concurrent metabolic, renal, and cardiovascular benefits [11,12,13]. Within this life-course CKMS framework, RSV is particularly well positioned for therapeutic consideration, given its capacity to restore redox homeostasis and modulate multiple signaling pathways relevant to CKMS-related disorders [14,15,16]. Thus, positioning RSV within a CKMS-centered life-course strategy reframes its role from adjunctive supplementation to a potential systems-level modulator of CKM health.

Emphasizing prevention over treatment in CKMS is essential because the syndrome evolves silently over decades through cumulative [17], self-reinforcing interactions among metabolic, renal, and cardiovascular pathways, many of which are programmed early in life [18,19,20]. The Developmental Origins of Health and Disease (DOHaD) framework highlights that adverse exposures during critical prenatal and early postnatal windows can permanently alter organ structure and function [21], predisposing individuals to insulin resistance, hypertension, nephron deficit, and cardiovascular vulnerability long before clinical disease emerges [22,23,24]. By the time CKMS becomes clinically apparent in adulthood, structural damage and functional adaptation in vulnerable organ systems—such as cardiovascular programming [22], kidney programming [23], and metabolic programming [24]—are often established and only partially reversible. Preventive strategies, namely reprogramming, enable intervention at early, subclinical stages, or even during developmental windows, when redox imbalance, inflammation, and metabolic dysregulation remain modifiable, yielding far greater long-term risk reduction [25]. A prevention-focused, life-course approach therefore shifts CKMS management from late-stage damage control to early trajectory modification, reducing cumulative disease burden, healthcare costs, and the intergenerational transmission of CKMS risk [26].

Regarding its antioxidant properties, resveratrol (RSV) functions primarily as a redox gene regulator rather than a direct in vivo free-radical scavenger [27]. It activates the Nrf2 (nuclear factor erythroid 2-related factor 2)–Antioxidant Response Element (ARE) axis to enhance endogenous antioxidant defenses, suppresses ROS generation by inhibiting NOX and optimizing mitochondrial function, and restores NO bioavailability [28,29]. In addition to these antioxidant effects, RSV exerts important cardiovascular actions through well-characterized signaling pathways.

A central mechanism involves activation of the SIRT1 (silent information regulator 1) pathway, which has been extensively reported as a key downstream mediator of resveratrol and interacts with AMPK (AMP-activated protein kinase) and PGC-1α to regulate cellular energy sensing, mitochondrial biogenesis, and stress resistance [30,31]. RSV also modulates Akt-dependent signaling and cardiac ion channel activity, mechanisms that contribute to the control of myocardial hypertrophy, electrophysiological stability, and vascular tone [32,33]. Concurrently, RSV suppresses NF-κB-mediated inflammation and limits mTOR (mechanistic target of rapamycin)-driven pathological remodeling [34,35], thereby attenuating fibrosis and adverse structural changes [36]. Moreover, RSV interacts bidirectionally with the gut microbiota by reshaping microbial composition while being metabolized by gut bacteria into bioactive derivatives that function as prebiotics [37]. Through these coordinated actions, RSV confers protection across the cardiovascular–kidney–metabolic axis by reducing vascular stiffness, limiting fibrosis, and preserving mitochondrial quality control. In the DOHaD framework, RSV acts as a reprogramming agent, and when administered during gestation or lactation, it can modulate key metabolic and signaling pathways in early life and prevent offspring from developing hypertension, obesity, and insulin resistance, all features belonging to CKMS [38,39].

**Figure 1:**
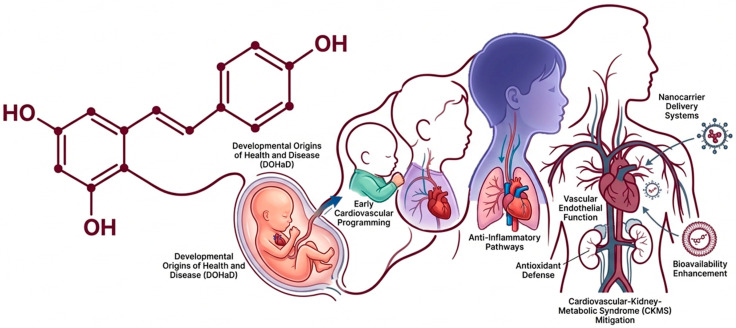
Resveratrol integrates cardiovascular–kidney–metabolic syndrome (CKMS) and life-course framework to modulate cardiovascular disease risk in later life.

Although clinical findings in humans remain heterogeneous owing to issues of dosing and bioavailability [40], compelling preclinical evidence supports RSV’s broad health benefits through integrated redox regulation, signaling modulation, and gut microbiota interactions. This narrative review synthesizes current evidence on RSV and cardiovascular health within an integrated CKMS and life-course framework, critically appraises both experimental and clinical data, and delineates key knowledge gaps that must be addressed to advance RSV from an experimental reprogramming agent to a clinically viable intervention with intergenerational relevance (Figure 1).

## 2. Materials and Methods

To accommodate the conceptual breadth of the field and the diversity of available evidence, we elected to conduct a narrative review that permits integrative interpretation across redox biology, nutritional science, pharmacology, developmental programming, and translational as well as preclinical research. The literature retrieval was performed through structured searches of MEDLINE, Embase, and the Cochrane Library restricted to English-language publications. The search framework was organized around interrelated themes encompassing resveratrol, oxidative stress, DOHaD, and CKMS, supplemented by manual examination of cited references to capture additional pertinent studies. Publications from January 2000 to December 2025 were considered for inclusion.

## 3. Cardiovascular Health Within an Integrated Life-Course Framework

Cardiovascular health is shaped by a lifelong trajectory beginning in utero, highlighting the importance of early-life determinants in shaping adult disease risk [41]. Integrating the heart, kidney, and metabolic systems within a life-course perspective underscores the need to shift public health strategies from reactive management of adult symptoms toward proactive prevention. CVD management must prioritize CKMS, as the cardiovascular, renal, and metabolic systems form a tightly interconnected, self-amplifying pathophysiological network in which dysfunction in one system accelerates injury in the others and ultimately drives CVD progression [4,5]. These conditions converge on shared mechanisms—including oxidative stress, NO deficiency, chronic inflammation, and gut dysbiosis [6,7,8,9,10]—supporting an integrated approach that enables earlier detection and holistic prevention rather than fragmented, organ-specific interventions. Given that many adult-onset disorders originate from early-life insults, embedding CKMS prevention within a life-course framework is essential for reducing global CVD mortality and disrupting the intergenerational transmission of risk.

### 3.1. The Integrated Biology of CKMS

The contemporary recognition of CKMS highlights the intricate interplay among cardiovascular, renal, and metabolic systems, encompassing disorders such as obesity, type 2 diabetes, CKD, and CVD. While staging frameworks (Stages 0–4) provide a structured approach for early prevention—ranging from primordial prevention at Stage 0 to overt clinical disease at Stage 4 [4,5]—they cannot fully capture the profound heterogeneity of disease trajectories across the life course [26]. Individuals with similar anthropometric profiles may diverge markedly in metabolic, renal, and cardiovascular outcomes due to nonlinear, bidirectional pathways shaped by genetic, developmental, behavioral, and social determinants. Effective CKMS prevention, therefore, requires shifting from late-stage risk stratification toward a life-course-oriented precision strategy that prioritizes the identification and modulation of underlying biological mechanisms during critical windows of developmental plasticity. By targeting these early, modifiable pathways, reprogramming interventions can redirect disease trajectories before irreversible CKMS is established, making mechanistic insight the cornerstone of early-life prevention [25,42].

### 3.2. Developmental Origins: The DOHaD Perspective

The DOHaD paradigm emphasizes that the fetal period represents a critical window of vulnerability, during which environmental exposures can shape long-term cardiovascular, renal, and metabolic outcomes. During these sensitive periods, structural and functional adaptations occur in response to maternal undernutrition [43], hypertensive disorders of pregnancy [44], gestational diabetes [45], environmental toxins [46], drug use [47], and other prenatal stressors [48], often leading to maladaptive programming that predisposes offspring to CKMS.

Historical events, such as the Dutch Hunger Winter of 1944–1945, provide compelling natural experiments demonstrating the lifelong impact of maternal malnutrition, with exposed offspring showing higher rates of obesity, type 2 diabetes, coronary artery disease, and hypertension in adulthood [36]. Longitudinal mother–child cohort studies further confirm that maternal nutritional status, metabolic disturbances, and illness are strong predictors of adult-onset CKMS in offspring [43,44,45,46,47,48].

Animal studies complement these findings, illustrating that early-life insults—including nutritional imbalance, maternal illness, placental or hypoxic stress, disruption of normal circadian rhythms, and exposure to drugs or toxins—can program cardiovascular, renal, and metabolic systems in offspring [49]. Such models are indispensable for dissecting the underlying mechanisms, as they allow controlled exploration of pathways conserved between humans and experimental systems. Key mechanistic drivers include oxidative stress, epigenetic dysregulation, disrupted nutrient-sensing signaling, aberrant renin–angiotensin system (RAS) activity, gut microbiota dysbiosis, chronic inflammation, and sex-specific effects [50,51,52,53,54]. Among these, oxidative stress emerges as a key regulatory mediator in the network, integrating diverse insults and amplifying susceptibility to CKMS across the lifespan [55].

### 3.3. Life-Course Trajectory of Redox Homeostasis

Oxidative stress arises when the generation of reactive oxygen species (ROS) exceeds the capacity of endogenous antioxidant defense systems [56]. It plays a central role in the developmental programming and progression of CKMS [57,58,59,60], exerting coordinated effects across multiple organs, including the kidneys [61], vasculature [62], and liver [63]. During normal pregnancy, physiological levels of ROS are essential for fetal growth and placental signaling; however, complicated pregnancies are frequently characterized by excessive oxidative stress [64]. Key mechanisms underlying oxidative stress in CKMS include upregulation of ROS-generating enzymes, increased ROS production, depletion of antioxidant reserves, accumulation of oxidative damage, and disruption of NO signaling pathways [65].

Oxidative damage can be assessed using well-established biomarkers, including 8-hydroxy-2′-deoxyguanosine (8-OHdG), F2-isoprostanes, malondialdehyde, 4-hydroxynonenal, and thiobarbituric acid-reactive substances [66,67,68,69,70]. Consistent with these pathways, experimental studies show that adverse developmental exposures are associated with increased reactive oxygen species (ROS) production and impaired antioxidant defenses, reflected by reduced activity of key enzymes such as superoxide dismutase (SOD), catalase (CAT), and glutathione peroxidase (GPx). These alterations have been observed after maternal nicotine exposure [62], glucocorticoid administration [66], postnatal overnutrition [67], and prenatal ethanol exposure [69].

Across the human life course, cardiovascular health evolves through distinct stages that are largely governed by the integrity of redox homeostasis [64,71]. During fetal life and childhood, antioxidant defenses are generally well-balanced against pro-oxidant forces, supporting normal cardiovascular and metabolic development [64]. Nevertheless, intracellular glutathione levels begin a gradual decline after early adulthood, marking an early shift toward increased redox vulnerability [72]. In midlife, the cumulative impact of biological aging, sedentary lifestyle, excess adiposity, and chronic physical or psychological stress commonly drives a progressive redox imbalance. This stage represents a critical window for lifestyle-based interventions aimed at preventing or delaying the emergence of CKM risk components. With advancing age, oxidative stress increasingly reflects the combined burden of mitochondrial dysfunction and established clinical risk factors, including hypertension, dyslipidemia, and diabetes [57,58,59,60]. Consequently, cardiovascular health is frequently compromised by overt clinical manifestations of the CKM cycle. Importantly, when oxidative stress originates in early life, these pathological trajectories may be accelerated, shifting CKMS onset and severity to earlier stages of life.

### 3.4. Reprogramming Cardiovascular Health: The Promise of Antioxidants

The concept of “reprogramming” suggests that the path toward CKM syndrome can be reversed or postponed by shifting interventions from adulthood to the earliest stages of life [25]. Within the DOHaD framework, antioxidant-based strategies have emerged as promising tools for reprogramming cardiovascular risk, particularly in the context of CKMS driven by oxidative stress [73,74,75]. Rather than merely counteracting oxidative damage at advanced disease stages, antioxidants hold potential to intervene earlier, restoring redox balance and altering pathological trajectories before irreversible structural and functional damage occurs [76].

Experimental studies provide compelling proof-of-concept that redox reprogramming is feasible. In diverse developmental programming models, antioxidant interventions during pregnancy and lactation—ranging from dietary polyphenols [77,78,79] and vitamins [80] to synthetic antioxidants (e.g., N-acetylcysteine) [81]—have been shown to normalize ROS levels, preserve NO bioavailability, and restore antioxidant enzyme activity in adult offspring. These effects translate into sustained improvements in vascular function, BP regulation, and metabolic homeostasis later in life [82,83].

## 4. Resveratrol

### 4.1. Synthesis and Sources

Resveratrol (RSV), a natural polyphenol, has emerged as a potent therapeutic and reprogramming agent due to its antioxidant properties and pleiotropic biofunctions [2,3,31,32]. RSV is synthesized by plants primarily as a defense response to environmental stressors, including mechanical injury, ultraviolet radiation, and microbial challenge [84]. In plant metabolism, resveratrol biosynthesis originates from glucose-derived precursors, where 4-coumaroyl-CoA condenses with malonyl-CoA through the action of stilbene synthase to generate *trans*-resveratrol. Under stress conditions, both *trans*- and *cis*-isomers may be formed, with *trans*-resveratrol representing the predominant and biologically active form in nature [84].

Natural dietary sources of resveratrol include Japanese knotweed, red grape varieties, and several berries. During food processing, particularly vinification, partial isomerization of *trans*-resveratrol to the *cis*-form can occur, influencing its stability and bioavailability. While *trans*-resveratrol remains relatively stable under light-protected and low-pH conditions, *cis*-resveratrol exhibits reduced stability, underscoring the importance of source and processing methods in determining final compound composition [85].

Beyond plant-derived sources, resveratrol can also be produced via chemical synthesis or microbial fermentation [86]. Yeast-based fermentation platforms have emerged as a scalable and standardized approach for producing high-purity trans-resveratrol, leading to the development of proposed monographs that define quality and safety parameters for its use as a food ingredient. Across these sources, resveratrol has demonstrated broad biological activities, including antimicrobial effects and modulation of gut microbiota composition, which collectively contribute to its reported cardiometabolic and anti-inflammatory benefits [87].

### 4.2. Metabolism

Resveratrol undergoes rapid and extensive metabolism following oral administration, which critically limits its systemic availability in the free form [88]. After passive diffusion or transporter-associated uptake across the intestinal epithelium, resveratrol is primarily subjected to phase II metabolism in the liver, where sulfation and glucuronidation represent the dominant metabolic pathways [89]. Consequently, circulating levels of unconjugated resveratrol remain very low [90].

In both plasma and target organs, resveratrol is detected predominantly as conjugated metabolites, including sulfate derivatives (*trans*-resveratrol-3,4′-disulfate, *trans*-resveratrol-3-sulfate, and *trans*-resveratrol-3,5-disulfate) and glucuronides (*trans*-resveratrol-4′-glucuronide and *trans*-resveratrol-3-glucuronide) [91]. Consistent with this rapid metabolic conversion, resveratrol exhibits a short elimination half-life of approximately 130–180 min [88]. In addition to these conjugates, resveratrol-derived metabolites such as piceatannol and dihydroresveratrol have also been identified in target tissues, suggesting the occurrence of secondary metabolic transformations [91,92].

Moreover, gut microbiota contribute to resveratrol metabolism by increasing its availability from dietary precursors and generating additional resveratrol derivatives [93]. Notably, substantial inter-individual variability has been reported, with oral absorption ranging from approximately 20–70% in humans and 15–50% in rats [94,95]. Collectively, these findings indicate that the bioavailability of resveratrol and its metabolites varies markedly and is largely determined by dose, administration regimen, and the intestinal microbial environment.

### 4.3. Resveratrol in Established CVD

In established CVD, RSV primarily targets molecular processes that drive disease progression, including oxidative stress, persistent inflammation, mitochondrial dysfunction, and maladaptive cardiac and vascular remodeling. Rather than acting as a direct antioxidant in vivo, resveratrol functions as a transcriptional regulator of redox homeostasis, enhancing endogenous antioxidant defenses while suppressing pathological ROS generation [96]. In parallel, inhibition of NF-κB-dependent inflammatory signaling and mTOR activity attenuates chronic inflammation and promotes autophagic clearance of damaged cellular components, mechanisms particularly relevant to failing myocardium and advanced atherosclerotic lesions [97]. Collectively, these actions restore endothelial NO bioavailability and improve vascular reactivity in diseased vessels.

Concurrently, resveratrol modulates key metabolic and stress-response pathways, notably SIRT1 and AMPK, thereby promoting mitochondrial biogenesis, metabolic flexibility, and cellular stress resilience in cardiomyocytes and endothelial cells [98,99]. Through these pleiotropic mechanisms, resveratrol shows potential as an adjunctive therapy for hypertension [100], atherosclerosis [101], endothelial dysfunction [102], stroke [103], and heart failure [104]. Future clinical translation will depend on optimized formulations, precise dosing strategies, and identification of patient subgroups most likely to benefit during advanced stages of CVD progression.

#### 4.3.1. Hypertension

In established hypertension, particularly when accompanied by vascular stiffness and endothelial dysfunction, resveratrol demonstrates disease-modifying potential rather than simple BP reduction [93]. Preclinical models consistently show improvement in endothelial nitric oxide synthase (eNOS) coupling, reduced vascular oxidative stress, and inhibition of vascular smooth muscle cell proliferation, translating into improved arterial compliance [105,106].

Human clinical studies evaluating the antihypertensive effects of resveratrol have yielded inconsistent results, with clinical trials investigating its impact on blood pressure reporting variable and sometimes conflicting findings [107,108]. In a systematic review and meta-analysis of 17 randomized controlled trials, resveratrol supplementation showed no significant overall effects on systolic, diastolic, mean blood pressure, or pulse pressure; however, modest systolic blood pressure reductions were observed in subgroups receiving high doses (≥300 mg/day) and in participants with diabetes. Resveratrol was generally well tolerated, supporting its potential adjunctive role in cardiovascular health when appropriately dosed and targeted [108].

In contrast, multiple trials—including those conducted in hypertensive or metabolically compromised populations—have shown no significant effects on SBP, diastolic blood pressure, or mean arterial pressure. Occasional adverse hemodynamic responses, such as increased diastolic pressure or heart rate, have also been reported. Overall, current human evidence remains inconclusive, but consistently indicates that future trials should employ adequately high doses and carefully defined hypertensive populations to clarify the therapeutic potential of resveratrol for blood pressure control.

#### 4.3.2. Atherosclerosis and Coronary Artery Disease

In established atherosclerosis, resveratrol modulates both lipid and inflammatory components of plaque progression by improving dyslipidemia, reducing LDL oxidation, and suppressing endothelial adhesion molecule expression, thereby limiting monocyte recruitment and foam cell formation [109]. Integrated transcriptomic and single-cell analyses highlight aging-related targets modulated by resveratrol, supporting its role in attenuating plaque progression [110]. Additionally, resveratrol inhibits platelet aggregation and enhances NO-mediated vasodilation, potentially reducing thrombotic risk [111]. Meta-analyses and clinical trials further demonstrate dose-dependent TNF-α reduction and increased eNOS expression, alongside improved vascular function and oxidative lipid profiles, indicating benefits in early atherosclerotic or high-risk patients [112].

#### 4.3.3. Stroke

RSV confers neuroprotection primarily by modulating redox balance and inflammatory signaling [113]. Preclinical studies show that RSV reduces infarct volume, cerebral edema, and neuronal damage via ischemic preconditioning, ER chaperone upregulation, and PINK1/Parkin-mediated mitophagy, while mitigating DJ-1 overoxidation [114]. Early clinical evidence suggests RSV co-administration with r-tPA improves neurological outcomes and long-term supplementation (100–200 mg/day) may reduce recurrent cerebrovascular events by enhancing endothelial function and lowering vascular stiffness [115]. Low oral bioavailability remains a challenge [83], prompting development of advanced formulations to improve brain delivery. Overall, RSV acts as a master regulator of antioxidant and anti-inflammatory pathways in stroke [116,117].

#### 4.3.4. Heart Failure and Pathological Cardiac Remodeling

In heart failure, resveratrol addresses core mechanisms driving disease progression, including pathological hypertrophy, fibrosis, and mitochondrial dysfunction [104]. By modulating transcriptional regulators associated with fetal gene reactivation and suppressing profibrotic signaling pathways, resveratrol attenuates adverse myocardial remodeling [118]. Activation of the SIRT1–PGC-1α axis improves mitochondrial quality control and energy production, critical for sustaining contractile function in failing myocardium [119].

Randomized clinical trials in patients with symptomatic heart failure have demonstrated improvements in systolic and diastolic function, exercise capacity, and inflammatory biomarkers [120], indicating that resveratrol can favorably influence both functional and molecular disease endpoints in established heart failure.

## 5. Translational Considerations of Resveratrol

### 5.1. Clinical Safety and Pharmacokinetic Barriers

RSV is generally well-tolerated at daily doses up to 1.0 g [121]. Doses exceeding 2.5 g/day frequently cause gastrointestinal side effects, including diarrhea, nausea, and abdominal pain [122]. Safety evaluation is complicated by RSV’s hormetic (biphasic) behavior: low doses act as protective antioxidants, whereas high doses can exert pro-oxidant effects and induce DNA damage [123]. Additionally, doses ≥ 1 g/day may inhibit cytochrome P450 enzymes, raising concerns regarding potential drug–drug interactions [124].

Successful clinical translation requires overcoming these pharmacokinetic barriers through advanced delivery systems. Strategies include utilizing lipid nanoparticles, liposomes, and polymeric micelles to bypass first-pass metabolism and improve stability [125,126]. Structural modifications, such as esterification with SCFAs (e.g., resveratrol–butyrate esters) [127], offer enhanced bioactivity and superior metabolic protection. Despite robust preclinical evidence, large-scale, standardized human randomized controlled trials are essential to define optimal dosing and validate RSV’s therapeutic potential across diverse populations.

Oral bioavailability of RSV is extremely low (<1%) due to rapid intestinal and hepatic glucuronidation and sulfation, limiting therapeutic tissue exposure. Overcoming these pharmacokinetic constraints is critical for successful clinical translation.

### 5.2. Advanced Delivery Strategies

Biomaterial-based delivery platforms aim to improve solubility, stability, intestinal absorption, controlled release, and tissue targeting. These strategies include lipid-based, polymer-based, inorganic, and hybrid nanocarriers.

#### 5.2.1. Lipid-Based Nanocarriers

Lipid-based delivery systems, including solid lipid nanoparticles (SLNs) and nanostructured lipid carriers (NLCs), enhance physicochemical stability, facilitate lymphatic uptake, and partially bypass first-pass metabolism, improving systemic exposure to resveratrol [128]. Surface functionalization further augments performance: for example, N-trimethyl chitosan–palmitic acid-coated SLNs increase oral bioavailability by approximately 3.8-fold through enhanced mucoadhesion and paracellular transport [129].

Liposomes, such as phospholipid bilayer vesicles, provide structural protection against premature enzymatic and chemical degradation while promoting cellular uptake. Chitosan-coated liposomes demonstrate superior in vitro antioxidant and anti-inflammatory activity in topical applications compared with free resveratrol [130].

PEGylated and pH-sensitive liposomes further improve therapeutic outcomes. PEGylation prolongs circulation time, enhances tumor accumulation via the enhanced permeability and retention (EPR) effect, and reduces resistance signaling in breast cancer models [131]. pH-sensitive formulations exploit acidic tumor microenvironments to achieve site-specific release, increasing therapeutic index and minimizing off-target effects [132].

#### 5.2.2. Polymer-Based Delivery Systems

Polymeric nanocarriers, including nanoparticles and micelles, offer a versatile platform for encapsulating hydrophobic compounds within stabilized core–shell structures. Polymeric micelles, typically 10–100 nm in diameter, substantially enhance the aqueous solubility of resveratrol and provide controlled, sustained release, thereby improving pharmacokinetic performance [133]. Among natural biopolymers, zein, a maize-derived prolamin protein, has received particular attention due to its biodegradability, biocompatibility, and ability to protect encapsulated compounds from acidic gastric conditions. Zein-based nanoparticles demonstrate enhanced stability under simulated gastrointestinal environments and improved oral bioavailability, while exhibiting reduced cytotoxicity compared with free resveratrol formulations [134].

Additionally, chitosan-coated nanoparticles leverage the cationic, mucoadhesive properties of chitosan to extend intestinal residence time and promote closer interaction with the epithelial surface, facilitating paracellular transport and enhancing systemic absorption [135].

#### 5.2.3. Inorganic and Hybrid Platforms

Cutting-edge research has expanded into inorganic nanocarriers and crystalline frameworks to maximize loading capacity and targeting. Mesoporous silica nanoparticles (MSNs) possess a honeycomb-like structure with high surface area and controllable pore diameters, allowing for the delivery of high concentrations of resveratrol [136]. Emerging platforms like Covalent Organic Frameworks (COFs) offer exceptional structural tunability and high porosity, enabling precise control over release kinetics via host–guest interactions [137]. Fluorinated COFs, for instance, have demonstrated superior drug retention and pH-responsive release profiles, which minimize off-target toxicity. Furthermore, hybrid systems integrating gold nanoparticles with resveratrol can be used for MRI-guided photothermal therapy, combining thermal ablation with molecular chemotherapy [138].

#### 5.2.4. Alternative Routes of Administration

Translational success may also depend on bypassing the gastrointestinal tract entirely through alternative administration routes. Inhalation therapy using dipalmitoylphosphatidylcholine-coated lipid nanoparticles (DPPC-LNs) has been proposed for site-specific treatment of pulmonary conditions, exhibiting a 48 h sustained release profile [139]. Transdermal delivery via microemulsions or nanostructured emulsions addresses various skin conditions while blocking the UV-dependent conversion of the active trans-isomer to the inactive cis-isomer [140]. For neurotherapeutic applications, chitosan-coated lipid microparticles have shown potential for direct nose-to-brain delivery, resulting in increased cerebrospinal fluid concentrations without systemic distribution [141]. Buccal delivery using mucoadhesive tablets also offers a localized treatment strategy for inflammatory oral lesions [142].

### 5.3. Chemical Modification Strategies

Chemical modifications complement delivery systems by improving metabolic stability, lipophilicity, and bioactivity [143,144]. Methoxylated analogues, including tetramethoxystilbene, exhibit enhanced metabolic stability, improved lipophilicity, and superior anticancer potency compared with native resveratrol, together with more favorable pharmacokinetic characteristics [144]. Prodrug approaches further address rapid phase II metabolism. For example, 3,5,4′-tri-O-acetylresveratrol (TARES) functions as a bio-reversible precursor that resists extensive intestinal conjugation, thereby increasing systemic exposure to free trans-resveratrol following in vivo deacetylation [145].

Integration of chemical modification with nano-encapsulation may synergistically enhance stability, tissue targeting, and therapeutic index. Future development depends on interdisciplinary approaches incorporating AI-guided nanomaterial design, pharmacogenomic stratification, and biomarker-driven clinical trials to address metabolic variability and optimize dosing [146].

### 5.4. Resveratrol–SCFA Ester Hybrids

Researchers have explored various structural modifications, including glycosylation, methylation, nanoformulation, and esterification with fatty acids [147]. These derivatives are designed to shield the molecule from premature enzymatic degradation and improve its distribution to target tissues. These derivatives have shown enhanced antioxidant, anti-inflammatory, and cardiometabolic effects in both in vitro and in vivo models [148,149]. Among these, esterification with SCFAs has shown particular promise within the context of developmental programming research [150]. SCFAs, such as acetate, propionate, and butyrate, are potent microbial metabolites that regulate metabolic and inflammatory pathways [151,152], making them ideal partners for molecular hybridization with RSV.

The synthesis of RSV–SCFA esters often utilizes Steglich esterification, a mild and efficient method that yields significantly higher products (73–82%) compared to traditional chemical techniques [153,154]. This process reacts trans-resveratrol with acetic acid, propionic acid, or n-butyric acid to produce mixtures of mono-, di-, and tri-esters. Specifically, these reactions yield resveratrol acetic acid ester (RAE), resveratrol propionic acid ester (RPE), and resveratrol butyric acid ester (RBE). Studies indicate that the antioxidant capacity of these esters is influenced by their chemical structure; for example, RAE exhibits superior lipid protection in corn oil models, while RPE shows the highest inhibition of LDL oxidation. Notably, all RSV–SCFA esters demonstrate stronger hydrogen peroxide scavenging activity than parent resveratrol [127].

The enhanced lipophilicity of RSV esters makes them better suited for incorporation into advanced drug delivery systems, such as lipid-based nanoparticles, liposomes, or polymeric micelles. These platforms protect against rapid metabolism and enable the sustained release of the compound. While these modified forms exhibit low toxicity and high efficacy in preclinical models, rigorous evaluation of dose equivalence and safety through human clinical trials remains essential to bridge the translational gap from laboratory findings to clinical practice.

## 6. Reprogramming Approach for Preventing CVD

Reprogramming strategies aim to prevent or reverse the long-term consequences of developmental programming before clinical manifestations of CVD arise. Because early CKMS programming contributes to later-life CVD, interventions are most effective during critical developmental windows, particularly gestation and early postnatal life, to prevent maladaptive programming in the cardiovascular [155], renal [156], and metabolic systems [157]. These strategies include nutritional modulation, pharmacological agents, physical activity, and microbiota-targeted therapies.

**Figure 2:**
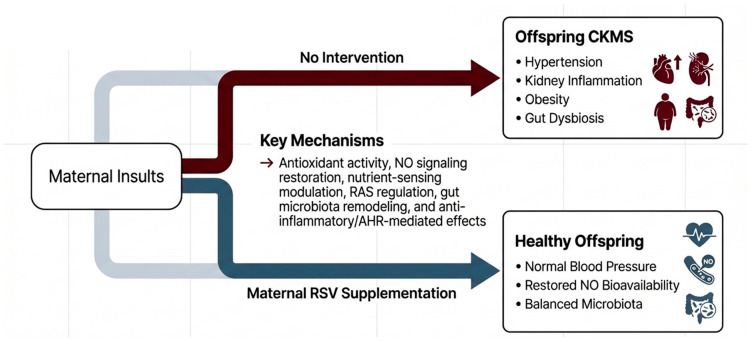
Maternal resveratrol supplementation as a preventive reprogramming strategy against offspring CKMS: underlying mechanisms and pathways.

Among these approaches, RSV and its derivatives play a central protective role against CKMS pathogenesis. Early-life RSV interventions have been extensively studied in preclinical models to guide potential translation to humans. RSV has demonstrated efficacy in mitigating key CKMS components, including hypertension, kidney dysfunction, metabolic disorders, and CVD [31,32]. This review specifically focuses on resveratrol and its derivatives administered during gestation and lactation as preventive reprogramming strategies against offspring CKMS (Figure 2). Each intervention is discussed in detail below.

### 6.1. Prevention of CKMS Programming by Resveratrol

Table 1 summarizes animal studies evaluating the efficacy of maternal resveratrol supplementation during gestation and lactation in preventing offspring CKMS [158,159,160,161,162,163,164,165,166,167,168,169,170]. Modeled early-life insults include maternal nutritional imbalances [158,164,165,167,168,169,170], maternal illness [159,166], and chemical exposures [161,163,164]. Resveratrol was most commonly administered via drinking water at 50 mg/L [158,159,160,161,162,163,164,165], with some studies using dietary supplementation (2–4 g/kg chow) [166,169], predominantly in rats and mice. Limited evidence from non-human primates suggests potential benefits on maternal and placental function and fetal liver development under Western-style diets [171,172], though long-term CKMS outcomes remain unknown.

Rodent studies consistently demonstrate that resveratrol exerts cardiovascular, renal, and metabolic protection through multiple mechanisms. Its antioxidant properties reduce renal oxidative stress, including 8-OHdG levels, and prevent hypertension induced by maternal CKD [159], 2,3,7,8-tetrachlorodibenzo-p-dioxin (TCDD) and dexamethasone [163], or Bisphenol A (BPA) combined with a high-fat diet [164]. By restoring NO bioavailability, resveratrol improves endothelial function and prevents hypertension across diverse models [159,160,163,164,166].

Hypertension programmed by maternal high-fat diet was associated with increased plasma angiotensin (Ang) I and reduced Ang (1–7) levels [165], which resveratrol reversed, while also lowering Ang II. In the maternal asymmetric dimethylarginine (ADMA) + trimethylamine-N-oxide (TMAO) exposure model, resveratrol prevented hypertension by downregulating ACE and AT1R while enhancing the non-classical RAS pathway [160].

Resveratrol also acts as a prebiotic modulator of the gut microbiome, reshaping microbial composition, increasing beneficial genera (e.g., *Bifidobacterium* and *Lactobacillus*), and enhancing diversity [158,159]. In high-fructose diet models, these microbial shifts were associated with lower blood pressure [158], while in L-NAME + high-fat-diet models, resveratrol decreased the *Firmicutes*-to-*Bacteroidetes* ratio, a marker linked to hypertension and kidney disease [162].

As a SIRT1 activator [173], resveratrol regulates AMPK and downstream PPAR target genes, which are critical for developmental programming of CKMS [174]. Consistently, resveratrol prevented hypertension in offspring exposed to maternal L-NAME + high-fat diet [162] or maternal high-fat diet alone [165].

Additionally, maternal exposure to TCDD or BPA activates the aryl hydrocarbon receptor (AHR) pathway [175,176], driving renal inflammation and T-cell-mediated cytokine accumulation [161,163,164]. Maternal resveratrol supplementation attenuated these effects by antagonizing AHR signaling and reducing offspring kidney inflammation.

Collectively, these studies indicate that maternal resveratrol supplementation prevents CKMS programming through antioxidant activity, NO signaling restoration, nutrient-sensing modulation, RAS regulation, gut microbiota remodeling, and anti-inflammatory/AHR-mediated effects, supporting its role as a multifaceted early-life reprogramming strategy.

### 6.2. Prevention of CKMS Programming by Resveratrol–SCFA Esters

Resveratrol analogues, including piceatannol [177] and trans-3,5,4′-trimethoxystilbene [178], have been reported to ameliorate several CKMS-related abnormalities. However, evidence regarding their use during gestation and lactation remains limited, and only a small number of studies have explored whether these compounds can modify developmental programming and thereby influence cardiometabolic outcomes in the offspring.

Among newly developed derivatives, resveratrol–SCFA esters (RBEs) have attracted attention because of their enhanced bioactivity and stability. Experimental studies using developmental exposure models of CKM risk have shown consistent protective effects. In a perinatal BPA exposure model, female offspring at 50 days of age exhibited increased body weight, hepatic lipid deposition, hyperlipidemia, and gut microbial imbalance after maternal BPA exposure; these alterations were markedly attenuated when RBEs were administered at 30 mg/kg/day during the perinatal period [179]. Similar protection was observed in male offspring, in whom RBE treatment reduced oxidative stress, prevented BPA-induced dysbiosis, and alleviated hepatic injury, accompanied by activation of antioxidant defense pathways and suppression of hepatic inflammation [180]. In addition, RBEs improved intestinal microbial profiles, increasing beneficial taxa such as S24-7 and *Adlercreutzia* and enhancing circulating SCFA concentrations, supporting a role in gut-mediated metabolic regulation [180].

The reprogramming potential of RBEs has also been evaluated in models of maternal exposure to endocrine-disrupting chemicals. In offspring born to dams exposed to di-2-ethylhexylphthalate (DEHP), elevated BP and increased body weight were evident at 12 weeks of age. Administration of high-dose RBE (6.67 mg/kg/day) during the perinatal period prevented both hypertension and excess weight gain, whereas a lower dose (3.33 mg/kg/day) produced only partial effects. Resveratrol at the same high dose prevented the rise in BP but did not normalize body weight, suggesting that esterification with SCFAs confers additional metabolic benefits. Mechanistic analyses indicated that RBEs attenuated oxidative stress, corrected gut microbial imbalance, and restored gut–kidney signaling through regulation of butyrate production and SCFA receptor pathways [181].

RBEs consist of several butyrate-conjugated resveratrol forms, including mono-, di-, and tri-butanoyl derivatives [127]. Among these, 3,4′-di-O-butanoylresveratrol (ED2) and 3-O-butanoylresveratrol (ED4) display particularly strong antioxidant capacity and have been examined in developmental programming models [127]. In offspring exposed to a maternal high-fructose diet, supplementation with ED2 or ED4 (25 mg/L in drinking water) throughout pregnancy and lactation significantly reduced the development of hypertension. ED2 enhanced antioxidant defenses and increased NO bioavailability while reshaping the gut microbiota, characterized by a higher abundance of *Bifidobacterium* and *Clostridium* and lower levels of *Angelakisella* and *Christensenella*. ED4, in contrast, predominantly influenced microbial metabolites, increasing SCFA production, upregulating SCFA receptor expression, and lowering circulating TMAO concentrations. These findings suggest that specific RBE derivatives may serve as effective perinatal interventions to counteract diet-induced cardiometabolic programming in the offspring [182].

### 6.3. Safety Concerns of Resveratrol Use During Pregnancy and Potential Offspring Consequences

Although resveratrol is widely recognized for its pleiotropic metabolic and cardioprotective properties, growing evidence highlights potential risks during pregnancy, particularly with respect to fetal development. Experimental studies suggest tissue-specific and dose-dependent adverse effects across species [183,184,185], raising concerns regarding gestational safety. Notably, the biological effects of resveratrol may vary according to developmental stage, and this stage-dependency may also extend to pathological conditions in which injured tissues re-activate fetal or neonatal gene programs, a phenomenon commonly observed in progressive cardiovascular, renal, and metabolic diseases. Such developmental reversion could modify tissue responsiveness to metabolic modulators, including resveratrol, and therefore warrants consideration when interpreting both experimental and clinical data.

An in vitro study using human fetal adrenocortical cells (9–12 weeks’ gestation) demonstrated that resveratrol (10 μM and 24 h) significantly suppressed ACTH-stimulated production of dehydroepiandrosterone, androstenedione, and 11-deoxycortisol through downregulation of CYP17 and CYP21, which are key enzymes in fetal adrenal steroidogenesis [186]. Given the critical role of adrenal steroids in fetal growth, sexual differentiation, and placental–fetal endocrine signaling, these findings suggest a potential risk of endocrine disruption and adverse long-term offspring outcomes. Accordingly, the investigators advised against resveratrol use during early pregnancy.

Despite over 100 randomized controlled trials (RCTs) reporting favorable cardiometabolic and anti-inflammatory effects in non-pregnant populations [187], clinical evidence in pregnant women—particularly regarding long-term offspring outcomes—remains lacking. Importantly, clinical testing in pregnant women requires not only well-designed human studies but also robust prior characterization of the toxicological and developmental safety profile in appropriate animal models, given the heightened vulnerability of the maternal–fetal unit. A systematic review of preclinical studies identified 31 reports (from 115 screened) across rodents, Japanese macaques, and sheep exposed to gestational resveratrol under various models of pregnancy complications, doses, and durations [188]. Maternal and fetal outcomes were highly heterogeneous and species-dependent, underscoring substantial translational uncertainty.

Most clinical trials to date have been small, short-term, and conducted in postmenopausal or metabolically impaired individuals, limiting extrapolation to pregnancy [187,189]. Therefore, clinical translation to pregnant populations requires consistent toxicological, pharmacokinetic, and developmental safety data from well-controlled animal studies. In addition, because diseased adult tissues may exhibit partial dedifferentiation toward fetal-like phenotypes, safety and efficacy findings obtained in developmental models should be interpreted together with data from disease models that mimic this reprogrammed state. Key issues—including safety, optimal dosing, placental transfer, developmental endocrine effects, and long-term cardiovascular–kidney–metabolic consequences in offspring—remain insufficiently characterized. Rigorous, adequately powered clinical trials with mechanistic endpoints and long-term offspring follow-up are essential to define the efficacy–safety balance of resveratrol supplementation during pregnancy.

## 7. Conclusions and Future Perspectives

**Figure 3:**
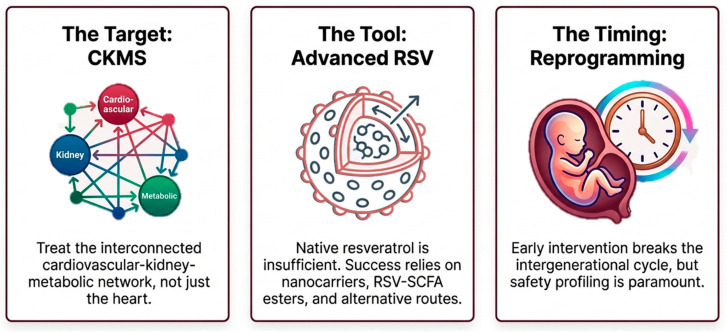
Life-course cardiovascular prevention with resveratrol integrates the CKMS network (Target), advanced RSV strategies (Tool), and early-life intervention windows (Timing) into a systems-based translational pathway.

The advancement of RSV from experimental promise to clinical implementation in CVD requires strategic realignment across three interconnected dimensions—target, tool, and timing (Figure 3).

First, the target must be conceptualized beyond isolated cardiac pathology and reframed within the integrated CKMS network. Oxidative stress, mitochondrial dysfunction, and NO dysregulation function as cross-organ amplifiers that propagate injury among cardiovascular, renal, and metabolic systems. A systems-biology approach is, therefore, essential, prioritizing network-based biomarkers, redox-sensitive signaling pathways, and multi-organ endpoints over single-organ surrogate outcomes. Such reframing aligns RSV research with contemporary precision cardiometabolic medicine.

Second, the tool requires technological evolution. Native RSV faces substantial translational barriers, including poor oral bioavailability, rapid first-pass metabolism, and extensive sulfation and glucuronidation that limit systemic exposure. Its hormetic dose–response profile further complicates clinical application, as antioxidant signaling at lower doses may shift toward pro-oxidant or toxic effects at higher concentrations [190]. Inconsistent trial outcomes in BP, glycemic control, and lipid metabolism likely reflect heterogeneity in formulations, pharmacokinetics, patient phenotypes, and study duration [191,192,193]. Addressing these limitations demands advanced delivery systems such as nanocarriers, structural analogues, RSV–SCFA esters, and alternative administration routes. Precision dosing strategies grounded in pharmacokinetic–pharmacodynamic modeling will be critical to standardize exposure and optimize therapeutic windows.

Third, the timing of intervention represents a pivotal determinant of efficacy. Preclinical evidence supports RSV as a developmental reprogramming agent capable of modifying early-life trajectories of CKMS risk within the DOHaD framework. However, robust human data remain limited, and rigorous safety profiling is essential, particularly during pregnancy and early postnatal life. Long-term, well-designed clinical trials incorporating pharmacokinetics, pharmacodynamics, validated redox biomarkers, and developmental safety endpoints are required to define life-course-specific therapeutic windows.

To support translational efforts, artificial intelligence (AI) may provide a complementary framework [194,195,196,197], but its application should be approached cautiously. Potential uses include integration of disease–gene databases (e.g., OMIM, GeneCards, and DisGeNET) [198,199,200] with drug–target repositories (e.g., TCMSP and DrugBank) [201,202], followed by protein–protein interaction mapping and pathway enrichment analyses (e.g., STRING and KEGG) [203,204]. However, all AI-derived hypotheses require rigorous experimental validation and careful assessment before clinical application, particularly when leveraging large independent human datasets (e.g., GEO) [205]. Deep learning algorithms, molecular docking simulations, and structural prediction platforms such as AlphaFold can assist in mechanistic exploration [206], but findings should be considered preliminary until independently confirmed.

Similarly, AI-assisted optimization of RSV formulation, extraction, and stability [207] should be regarded as a tool for hypothesis generation rather than definitive evidence. Future applications, such as digital twins or in silico clinical trials, hold promise for simulating RSV interventions across diverse populations [208], but these approaches require validation and careful interpretation. AI-guided molecular design of RSV derivatives may enable context-dependent modulation between antioxidant and pro-oxidant signaling [195,209], yet all predictions must be experimentally corroborated.

Integrating AI with rigorous experimental and clinical investigation establishes a cautious life-course translational framework for RSV. This systems-oriented strategy incorporates standardized endpoints, validated biomarkers, pharmacogenomic stratification, and developmental timing considerations to enhance therapeutic precision across cardiovascular, renal, and metabolic systems. Through coordinated innovation in target definition, technological refinement, and intervention timing, RSV can transition from a promising redox modulator to a clinically viable, precision-guided intervention for durable cardiovascular protection across the lifespan, with AI serving as a supportive, but rigorously validated tool rather than a definitive solution.

## Figures and Tables

**Table 1:** Animal studies representing how perinatal resveratrol prevents offspring CKMS.

CKMS Component	Animal Model (Species)	Resveratrol Dose/Timing	Reprogramming Effects and Mechanisms	Ref.
Cardiovascular/Renal	Maternal and post-weaning high-fructose diet (rats)	50 mg/L in water (G/L)	Prevention of hypertension, reduction in oxidative stress, activation of nutrient-sensing pathways, and modulation of gut microbiota	[158]
Cardiovascular/Renal	Maternal chronic kidney disease (rats)	50 mg/L in water (G/L)	Prevention of hypertension, remodeling the gut microbiota, modulation of SCFA signaling, improvement of NO pathways, and reduction in oxidative stress	[159]
Cardiovascular/Renal	Maternal ADMA + TMAO exposure (rats)	50 mg/L in water (G/L)	Prevention of hypertension, restoration of NO bioavailability, remodeling of gut microbiota, enhancement of SCFA production, and regulation of the RAS	[160]
Cardiovascular/Renal	Maternal TCDD exposure (rats)	50 mg/L in water (G/L)	Prevention of hypertension, antagonizing AHR signaling, suppression of TH17-mediated renal inflammation, and reshaping gut microbiota composition	[161]
Cardiovascular/Renal	Maternal L-NAME + high-fat diet (rats)	50 mg/L in water (G/L)	Prevention of hypertension, reduction in oxidative stress, restoration of AMPK/PGC-1α nutrient-sensing signaling, and reshaping gut microbiota composition	[162]
Cardiovascular/Renal	Maternal TCDD + prenatal dexamethasone exposure (rats)	50 mg/L in water (G/L)	Prevention of hypertension, reduction in oxidative stress, restoration of NO bioavailability, antagonizing AHR signaling, and suppression of the RAS	[163]
Cardiovascular/Renal	Bisphenol A + high-fat diet (rats)	50 mg/L in water (G/L)	Prevention of hypertension, reduction in oxidative stress, restoration of NO bioavailability, and antagonizing AHR signaling	[164]
Cardiovascular/Renal	Maternal high-fat diet (rats)	50 mg/L in water (G/L)	Prevention of hypertension, reduction in oxidative stress, rebalance of the RAS, and restoration of AMPK–PGC-1α nutrient-sensing signaling	[165]
Cardiovascular	Maternal hypertension (rats)	4 g/kg in diet (G/L)	Prevention of hypertension and restoration of NO bioavailability	[166]
Metabolic	Maternal high-fat diet (rats)	50 mg/L in water (G/L)	Improvement of metabolic parameters, including adiposity, dyslipidemia, hyperleptinemia, and glucose intolerance, restoration of SIRT1 signaling, rebalance of the RAS	[167]
Metabolic	Maternal high-fat diet (rats)	50 mg/L in water (G/L)	Attenuation of adiposity, visceral and subcutaneous fat accumulation, and hyperleptinemia	[168]
Metabolic	Maternal high-fat diet (mice)	2 g/kg in diet (G/L)	Protection against obesity and metabolic dysfunction	[169]
Metabolic	Maternal protein restriction (rats)	25 mg/kg/day (G)	Protection against metabolic dysfunction and reduction in oxidative stress	[170]

ADMA = asymmetric dimethylarginine; TMAO = trimethylamine-N-oxide; TCDD = 2,3,7,8-tetrachlorodibenzo-p-dioxin; L-NAME = N^G^-nitro-L-arginine-methyl ester; G = gestation; L = lactation; SCFA = short-chain fatty acid; NO = nitric oxide; RAS = renin–angiotensin system; AHR = aryl hydrocarbon receptor.

## Data Availability

No new data were created or analyzed in this study. Data sharing is not applicable to this article.

## References

[1] Singla R.K., Dubey A.K., Garg A., Sharma R.K., Fiorino M., Ameen S.M., Haddad M.A., Al-Hiary M. (2019). Natural Polyphenols: Chemical Classification, Definition of Classes, Subcategories, and Structures. J. AOAC Int..

[2] Singh A.P., Singh R., Verma S.S., Rai V., Kaschula C.H., Maiti P., Gupta S.C. (2019). Health benefits of resveratrol: Evidence from clinical studies. Med. Res. Rev..

[3] Frémont L. (2000). Biological effects of resveratrol. Life Sci..

[4] Ndumele C.E., Neeland I.J., Tuttle K.R., Chow S.L., Mathew R.O., Khan S.S., Coresh J., Baker-Smith C.M., Carnethon M.R., Després J.P. (2023). A Synopsis of the Evidence for the Science and Clinical Management of Cardiovascular-Kidney-Metabolic (CKM) Syndrome: A Scientific Statement From the American Heart Association. Circulation.

[5] Kittelson K.S., Junior A.G., Fillmore N., da Silva Gomes R. (2024). Cardiovascular-kidney-metabolic syndrome—An integrative review. Prog. Cardiovasc. Dis..

[6] Thompson L.P., Al-Hasan Y. (2012). Impact of oxidative stress in fetal programming. J. Pregnancy.

[7] Xu Z., Yang S., Tan Y., Zhang Q., Wang H., Tao J., Liu Q., Wang Q., Feng W., Li Z. (2025). Inflammation in cardiovascular-kidney-metabolic syndrome: Key roles and underlying mechanisms-a comprehensive review. Mol. Cell. Biochem..

[8] Liao G.Z., He C.H., Zhang Y.H., Zhang J. (2025). Addressing the “Nitric Oxide Crisis” in Cardiovascular-Kidney-Metabolic Syndrome: Therapeutic Potential of the Inorganic Nitrate-Nitrite-NO Pathway. Obes. Rev..

[9] Tang W.H., Kitai T., Hazen S.L. (2017). Gut Microbiota in Cardiovascular Health and Disease. Circ. Res..

[10] Bloom P.P., Garrett W.S., Penniston K.L., Winkler M.H., Hazen S.L., Agudelo J., Suryavanshi M., Babiker A., Dodd D., Fischbach M.A. (2025). Microbiota and kidney disease: The road ahead. Nat. Rev. Nephrol..

[11] Duan L., Yang H., Chen Z., Zhao J., Yang J., Cai D. (2025). Dietary antioxidants and mortality in early-stage CKM syndrome: Insights from NHANES. Nutr. Metab..

[12] Sharebiani H., Mokaram M., Mirghani M., Fazeli B., Stanek A. (2024). The Effects of Antioxidant Supplementation on the Pathologic Mechanisms of Metabolic Syndrome and Cardiovascular Disease Development. Nutrients.

[13] Liu H., Jiao Y., Wang P.C., Chen Y., Xu M., Zhang X., Zheng X., Yang Z. (2026). Oxidative stress and antioxidant therapeutic mechanisms. Pharmacol. Ther..

[14] Gál R., Halmosi R., Gallyas F., Tschida M., Mutirangura P., Tóth K., Alexy T., Czopf L. (2023). Resveratrol and beyond: The Effect of Natural Polyphenols on the Cardiovascular System: A Narrative Review. Biomedicines.

[15] Asgary S., Karimi R., Momtaz S., Naseri R., Farzaei M.H. (2019). Effect of resveratrol on metabolic syndrome components: A systematic review and meta-analysis. Rev. Endocr. Metab. Disord..

[16] Den Hartogh D.J., Tsiani E. (2019). Health Benefits of Resveratrol in Kidney Disease: Evidence from In Vitro and In Vivo Studies. Nutrients.

[17] Jaradat J.H., Nashwan A.J. (2023). Cardiovascular-kidney-metabolic syndrome: Understanding the interconnections and the need for holistic intervention. J. Med. Surg. Public Health.

[18] Nüsken E., Dötsch J., Weber L.T., Nüsken K.D. (2018). Developmental Programming of Renal Function and Re-Programming Approaches. Front. Pediatr..

[19] Ma N., Hardy D.B. (2012). The Fetal Origins of the Metabolic Syndrome: Can We Intervene?. J. Pregnancy.

[20] Thornburg K.L. (2015). The programming of cardiovascular disease. J. Dev. Orig. Health Dis..

[21] Fleming T.P., Velazquez M.A., Eckert J.J. (2015). Embryos, DOHaD and David Barker. J. Dev. Orig. Health Dis..

[22] Blackmore H.L., Ozanne S.E. (2015). Programming of cardiovascular disease across the life-course. J. Mol. Cell. Cardiol..

[23] Kett M.M., Denton K.M. (2011). Renal programming: Cause for concern?. Am. J. Physiol. Regul. Integr. Comp. Physiol..

[24] Fall C.H.D., Kumaran K. (2019). Metabolic programming in early life in humans. Philos. Trans. R. Soc. Lond. B Biol. Sci..

[25] Paauw N.D., van Rijn B.B., Lely A.T., Joles J.A. (2017). Pregnancy as a critical window for blood pressure regulation in mother and child: Programming and reprogramming. Acta Physiol..

[26] Khan S.S., Coresh J., Pencina M.J., Ndumele C.E., Rangaswami J., Chow S.L., Palaniappan L.P., Sperling L.S., Virani S.S., Ho J.E. (2023). Novel Prediction Equations for Absolute Risk Assessment of Total Cardiovascular Disease Incorporating Cardiovascular-Kidney-Metabolic Health: A Scientific Statement From the American Heart Association. Circulation.

[27] Oh W.Y., Shahidi F. (2018). Antioxidant activity of resveratrol ester derivatives in food and biological model systems. Food Chem..

[28] Kulkarni S.S., Cantó C. (2015). The molecular targets of resveratrol. Biochim. Biophys. Acta.

[29] Abolfazli S., Karav S., Johnston T.P., Sahebkar A. (2025). Regulatory effects of resveratrol on nitric oxide signaling in cardiovascular diseases. Pharmacol. Rep..

[30] Price N.L., Gomes A.P., Ling A.J., Duarte F.V., Martin-Montalvo A., North B.J., Agarwal B., Ye L., Ramadori G., Teodoro J.S. (2012). SIRT1 is required for AMPK activation and the beneficial effects of resveratrol on mitochondrial function. Cell Metab..

[31] Lagouge M., Argmann C., Gerhart-Hines Z., Meziane H., Lerin C., Daussin F., Messadeq N., Milne J., Lambert P., Elliott P. (2006). Resveratrol improves mitochondrial function and protects against metabolic disease by activating SIRT1 and PGC-1alpha. Cell.

[32] Baczkó I., Light P.E. (2015). Resveratrol and derivatives for the treatment of atrial fibrillation. Ann. N. Y. Acad. Sci..

[33] Chan A.Y., Dolinsky V.W., Soltys C.L., Viollet B., Baksh S., Light P.E., Dyck J.R. (2008). Resveratrol inhibits cardiac hypertrophy via AMP-activated protein kinase and Akt. J. Biol. Chem..

[34] Ma E., Wu C., Chen J., Wo D., Ren D.N., Yan H., Peng L., Zhu W. (2023). Resveratrol prevents Ang II-induced cardiac hypertrophy by inhibition of NF-κB signaling. Biomed. Pharmacother..

[35] Guan P., Sun Z.M., Wang N., Zhou J., Luo L.F., Zhao Y.S., Ji E.S. (2019). Resveratrol prevents chronic intermittent hypoxia-induced cardiac hypertrophy by targeting the PI3K/AKT/mTOR pathway. Life Sci..

[36] Strunz C.M.C., Roggerio A., Cruz P.L., Benvenuti L.A., Irigoyen M.C., Mansur A.P. (2025). Resveratrol Attenuates Fibrosis and Alters Signaling Pathways in Diabetic Cardiac and Skeletal Muscles and Adipose Tissue Without Reversing Structural Damage. Int. J. Mol. Sci..

[37] Inchingolo A.D., Malcangi G., Inchingolo A.M., Piras F., Settanni V., Garofoli G., Palmieri G., Ceci S., Patano A., De Leonardis N. (2022). Benefits and Implications of Resveratrol Supplementation on Microbiota Modulations: A Systematic Review of the Literature. Int. J. Mol. Sci..

[38] Hsu C.N., Hou C.Y., Tain Y.L. (2021). Preventive Aspects of Early Resveratrol Supplementation in Cardiovascular and Kidney Disease of Developmental Origins. Int. J. Mol. Sci..

[39] Tain Y.L., Hsu C.N. (2018). Developmental Programming of the Metabolic Syndrome: Can We Reprogram with Resveratrol?. Int. J. Mol. Sci..

[40] De Vries K., Strydom M., Steenkamp V. (2018). Bioavailability of resveratrol: Possibilities for enhancement. J. Herb. Med..

[41] Falkner B., Gidding S. (2021). Life-Course Implications of Pediatric Risk Factors for Cardiovascular Disease. Can. J. Cardiol..

[42] Giugni F.R., Berry J.D., Khera A., Shah A.M., de Lemos J.A. (2024). Precision Medicine for Cardiovascular Prevention and Population Health: A Bridge Too Far?. Circulation.

[43] Roseboom T., de Rooij S., Painter R. (2006). The Dutch famine and its long-term consequences for adult health. Early Hum. Dev..

[44] Fraser A., Nelson S.M., Macdonald-Wallis C., Sattar N., Lawlor D.A. (2013). Hypertensive disorders of pregnancy and cardiometabolic health in adolescent offspring. Hypertension.

[45] Moon J.H., Jang H.C. (2022). Gestational Diabetes Mellitus: Diagnostic Approaches and Maternal-Offspring Complications. Diabetes Metab. J..

[46] Hsu C.N., Tain Y.L. (2021). Adverse Impact of Environmental Chemicals on Developmental Origins of Kidney Disease and Hypertension. Front. Endocrinol..

[47] Schreuder M.F., Bueters R.R., Huigen M.C., Russel F.G., Masereeuw R., van den Heuvel L.P. (2011). Effect of drugs on renal development. Clin. J. Am. Soc. Nephrol..

[48] Hsu C.N., Tain Y.L. (2020). Light and Circadian Signaling Pathway in Pregnancy: Programming of Adult Health and Disease. Int. J. Mol. Sci..

[49] Tain Y.L., Lin Y.J., Hsu C.N. (2025). Animal Models for Studying Developmental Origins of Cardiovascular-Kidney-Metabolic Syndrome. Biomedicines.

[50] Padmanabhan V., Cardoso R.C., Puttabyatappa M. (2016). Developmental Programming, a Pathway to Disease. Endocrinology.

[51] Sandman C.A., Glynn L.M., Davis E.P. (2013). Is there a viability-vulnerability tradeoff? Sex differences in fetal programming. J. Psychosom. Res..

[52] Lee M.A., Böhm M., Paul M., Ganten D. (1993). Tissue renin-angiotensin systems. Their role in cardiovascular disease. Circulation.

[53] Hsu C.N., Lin Y.J., Hou C.Y., Chen Y.W., Chang-Chien G.P., Lin S.F., Tain Y.L. (2025). Antioxidants, Gut Microbiota, and Cardiovascular Programming: Unraveling a Triad of Early-Life Interactions. Antioxidants.

[54] Bianco-Miotto T., Craig J.M., Gasser Y.P., van Dijk S.J., Ozanne S.E. (2017). Epigenetics and DOHaD: From basics to birth and beyond. J. Dev. Orig. Health Dis..

[55] Pizzino G., Irrera N., Cucinotta M., Pallio G., Mannino F., Arcoraci V., Squadrito F., Altavilla D., Bitto A. (2017). Oxidative Stress: Harms and Benefits for Human Health. Oxid. Med. Cell. Longev..

[56] Sies H. (2015). Oxidative stress: A concept in redox biology and medicine. Redox Biol..

[57] Spahis S., Borys J.M., Levy E. (2017). Metabolic Syndrome as a Multifaceted Risk Factor for Oxidative Stress. Antioxid. Redox Signal..

[58] Ravarotto V., Simioni F., Pagnin E., Davis P.A., Calò L.A. (2018). Oxidative stress-chronic kidney disease-cardiovascular disease: A vicious circle. Life Sci..

[59] Henriksen E.J., Diamond-Stanic M.K., Marchionne E.M. (2011). Oxidative stress and the etiology of insulin resistance and type 2 diabetes. Free Radic. Biol. Med..

[60] Wilcox C.S. (2005). Oxidative stress and nitric oxide deficiency in the kidney: A critical link to hypertension?. Am. J. Physiol. Regul. Integr. Comp. Physiol..

[61] Tain Y.L., Hsieh C.S., Lin I.C., Chen C.C., Sheen J.M., Huang L.T. (2010). Effects of maternal L-citrulline supplementation on renal function and blood pressure in offspring exposed to maternal caloric restriction: The impact of nitric oxide pathway. Nitric Oxide.

[62] Xiao D., Huang X., Yang S., Zhang L. (2011). Antenatal nicotine induces heightened oxidative stress and vascular dysfunction in rat offspring. Br. J. Pharmacol..

[63] Conceição E.P., Peixoto-Silva N., Pinheiro C.R., Oliveira E., Moura E.G., Lisboa P.C. (2015). Maternal nicotine exposure leads to higher liver oxidative stress and steatosis in adult rat offspring. Food Chem. Toxicol..

[64] Dennery P.A. (2010). Oxidative stress in development: Nature or nurture?. Free Radic. Biol. Med..

[65] Wilcox C.S., Gutterman D. (2005). Focus on oxidative stress in the cardiovascular and renal systems. Am. J. Physiol. Heart Circ. Physiol..

[66] Lamothe J., Khurana S., Tharmalingam S., Williamson C., Byrne C.J., Lees S.J., Khaper N., Kumar A., Tai T.C. (2021). Oxidative Stress Mediates the Fetal Programming of Hypertension by Glucocorticoids. Antioxidants.

[67] Conceição E.P., Franco J.G., Oliveira E., Resende A.C., Amaral T.A., Peixoto-Silva N., Passos M.C., Moura E.G., Lisboa P.C. (2013). Oxidative stress programming in a rat model of postnatal early overnutrition—Role of insulin resistance. J. Nutr. Biochem..

[68] Ojeda N.B., Hennington B.S., Williamson D.T., Hill M.L., Betson N.E., Sartori-Valinotti J.C., Reckelhoff J.F., Royals T.P., Alexander B.T. (2012). Oxidative stress contributes to sex differences in blood pressure in adult growth-restricted offspring. Hypertension.

[69] Contreras M.L., de la Fuente-Ortega E., Vargas-Roberts S., Muñoz D.C., Goic C.A., Haeger P.A. (2017). NADPH Oxidase Isoform 2 (NOX2) Is Involved in Drug Addiction Vulnerability in Progeny Developmentally Exposed to Ethanol. Front. Neurosci..

[70] Martínez Gascón L.E., Ortiz M.C., Galindo M., Sanchez J.M., Sancho-Rodriguez N., Albaladejo Otón M.D., Rodriguez Mulero M.D., Rodriguez F. (2022). Role of heme oxygenase in the regulation of the renal hemodynamics in a model of sex dependent programmed hypertension by maternal diabetes. Am. J. Physiol. Regul. Integr. Comp. Physiol..

[71] Finkel T., Holbrook N.J. (2000). Oxidants, oxidative stress and the biology of ageing. Nature.

[72] Maher P. (2005). The effects of stress and aging on glutathione metabolism. Ageing Res. Rev..

[73] Hsu C.N., Tain Y.L. (2021). Developmental Origins of Kidney Disease: Why Oxidative Stress Matters?. Antioxidants.

[74] Rodríguez-Rodríguez P., Ramiro-Cortijo D., Reyes-Hernández C.G., López de Pablo A.L., González M.C., Arribas S.M. (2018). Implication of Oxidative Stressin Fetal Programming of Cardiovascular Disease. Front. Physiol..

[75] Tain Y.L., Hsu C.N. (2022). Metabolic Syndrome Programming and Reprogramming: Mechanistic Aspects of Oxidative Stress. Antioxidants.

[76] Sebastiani G., Navarro-Tapia E., Almeida-Toledano L., Serra-Delgado M., Paltrinieri A.L., García-Algar Ó., Andreu-Fernández V. (2022). Effects of Antioxidant Intake on Fetal Development and Maternal/Neonatal Health during Pregnancy. Antioxidants.

[77] Liang C., Oest M.E., Prater M.R. (2009). Intrauterine exposure to high saturated fat diet elevates risk of adult-onset chronic diseases in C57BL/6 mice. Birth Defects Res. B Dev. Reprod. Toxicol..

[78] Sato S., Norikura T., Mukai Y. (2019). Maternal quercetin intake during lactation attenuates renal inflammation and modulates autophagy flux in high-fructose-diet-fed female rat offspring exposed to maternal malnutrition. Food Funct..

[79] Hsu C.N., Hou C.Y., Chang-Chien G.P., Lin S., Tain Y.L. (2021). Maternal Garlic Oil Supplementation Prevents High-Fat Diet-Induced Hypertension in Adult Rat Offspring: Implications of H2S-Generating Pathway in the Gut and Kidneys. Mol. Nutr. Food Res..

[80] Wang J., Yin N., Deng Y., Wei Y., Huang Y., Pu X., Li L., Zheng Y., Guo J., Yu J. (2016). Ascorbic Acid Protects against Hypertension through Downregulation of ACE1 Gene Expression Mediated by Histone Deacetylation in Prenatal Inflammation-Induced Offspring. Sci. Rep..

[81] Tain Y.L., Hsu C.N., Lee C.T., Lin Y.J., Tsai C.C. (2016). N-Acetylcysteine prevents programmed hypertension in male rat offspring born to suramin-treated mothers. Biol. Reprod..

[82] Tain Y.L., Hsu C.N. (2022). Oxidative Stress-Induced Hypertension of Developmental Origins: Preventive Aspects of Antioxidant Therapy. Antioxidants.

[83] Tain Y.L., Hsu C.N. (2024). Maternal Polyphenols and Offspring Cardiovascular-Kidney-Metabolic Health. Nutrients.

[84] Mei Y.Z., Liu R.X., Wang D.P., Wang X., Dai C.C. (2015). Biocatalysis and biotransformation of resveratrol in microorganisms. Biotechnol. Lett..

[85] Fabjanowicz M., Płotka-Wasylka J., Namies’nik J. (2018). Detection, identifification and determination of resveratrol in wine. Problems and challenges. TrAC Trends Anal. Chem..

[86] Bostanghadiri N., Pormohammad A., Chirani A.S., Pouriran R., Erfanimanesh S., Hashemi A. (2017). Comprehensive review on the antimicrobial potency of the plant polyphenol Resveratrol. Biomed. Pharmacother..

[87] Shen L., Ji H.F. (2018). Reciprocal Interactions Between Resveratrol and Gut Microbiota Deepen Our Understanding of Molecular Mechanisms Underlying Its Health Benefifits. Trends Food Sci. Technol..

[88] Gambini J., Inglés M., Olaso G., Lopez-Grueso R., Bonet-Costa V., Gimeno-Mallench L., Mas-Bargues C., Abdelaziz K.M., Gomez-Cabrera M.C., Vina J. (2015). Properties of resveratrol: In vitro and in vivo studies about metabolism, bioavailability, and biological effects in animal models and humans. Oxid. Med. Cell. Longev..

[89] Yu C., Shin Y.G., Chow A., Li Y., Kosmeder J.W., Lee Y.S., Hirschelman W.H., Pezzuto J.M., Mehta R.G., van Breemen R.B. (2002). Human, rat, and mouse metabolism of resveratrol. Pharm. Res..

[90] Walle T. (2011). Bioavailability of resveratrol. Ann. N. Y. Acad. Sci..

[91] Menet M.C., Baron S., Taghi M., Diestra R., Dargère D., Laprévote O., Nivet-Antoine V., Beaudeux J.L., Bédarida T., Cottart C.H. (2017). Distribution of trans-resveratrol and its metabolites after acute or sustained administration in mouse heart, brain, and liver. Mol. Nutr. Food Res..

[92] Potter G.A., Patterson L.H., Wanogho E., Perry P.J., Butler P.C., Ijaz T., Ruparelia K.C., Lamb J.H., Farmer P.B., Stanley L.A. (2002). The cancer preventative agent resveratrol is converted to the anticancer agent piceatannol by the cytochrome P450 enzyme CYP1B1. Br. J. Cancer.

[93] Chaplin A., Carpéné C., Mercader J. (2018). Resveratrol, Metabolic Syndrome, and Gut Microbiota. Nutrients.

[94] Walle T., Hsieh F., DeLegge M.H., Oatis J.E., Walle U.K. (2004). High absorption but very low bioavailability of oral resveratrol in humans. Drug Metab. Dispos..

[95] Marier J.F., Vachon P., Gritsas A., Zhang J., Moreau J.P., Ducharme M.P. (2002). Metabolism and disposition of resveratrol in rats: Extent of absorption, glucuronidation, and enterohepatic recirculation evidenced by a linked-rat model. J. Pharmacol. Exp. Ther..

[96] Xia N., Daiber A., Förstermann U., Li H. (2017). Antioxidant effects of resveratrol in the cardiovascular system. Br. J. Pharmacol..

[97] Teimouri M., Homayouni-Tabrizi M., Rajabian A., Amiri H., Hosseini H. (2022). Anti-inflammatory effects of resveratrol in patients with cardiovascular disease: A systematic review and meta-analysis of randomized controlled trials. Complement. Ther. Med..

[98] Carrizzo A., Puca A., Damato A., Marino M., Franco E., Pompeo F., Traficante A., Civitillo F., Santini L., Trimarco V. (2013). Resveratrol improves vascular function in patients with hypertension and dyslipidemia by modulating NO metabolism. Hypertension.

[99] Csiszar A., Labinskyy N., Pinto J.T., Ballabh P., Zhang H., Losonczy G., Pearson K., de Cabo R., Pacher P., Zhang C. (2009). Resveratrol induces mitochondrial biogenesis in endothelial cells. Am. J. Physiol. Heart Circ. Physiol..

[100] DiNatale J.C., Crowe-White K.M. (2022). Effects of resveratrol supplementation on nitric oxide-mediated vascular outcomes in hypertension: A systematic review. Nitric Oxide.

[101] Guo S., Zhou Y., Xie X. (2022). Resveratrol inhibiting TGF/ERK signaling pathway can improve atherosclerosis: Backgrounds, mechanisms and effects. Biomed. Pharmacother..

[102] Parsamanesh N., Asghari A., Sardari S., Tasbandi A., Jamialahmadi T., Xu S., Sahebkar A. (2021). Resveratrol and endothelial function: A literature review. Pharmacol. Res..

[103] Nabavi S.F., Li H., Daglia M., Nabavi S.M. (2014). Resveratrol and stroke: From chemistry to medicine. Curr. Neurovasc. Res..

[104] Dyck G.J.B., Raj P., Zieroth S., Dyck J.R.B., Ezekowitz J.A. (2019). The Effects of Resveratrol in Patients with Cardiovascular Disease and Heart Failure: A Narrative Review. Int. J. Mol. Sci..

[105] Behbahani J., Thandapilly S.J., Louis X.L., Huang Y., Shao Z., Kopilas M.A., Wojciechowski P., Netticadan T., Anderson H.D. (2010). Resveratrol and small artery compliance and remodeling in the spontaneously hypertensive rat. Am. J. Hypertens..

[106] Breen D.M., Dolinsky V.W., Zhang H., Ghanim H., Guo J., Mroziewicz M., Tsiani E.L., Bendeck M.P., Dandona P., Dyck J.R. (2012). Resveratrol inhibits neointimal formation after arterial injury through an endothelial nitric oxide synthase-dependent mechanism. Atherosclerosis.

[107] Shafiei E., Rezaei M., Mahmoodi M., Amini A., Hajian N., Sanjideh Z., Alizadeh M., Farrokhi S., Smoliga J., Raj P. (2025). Preliminary, randomized, double-blinded, placebo-controlled cross-over study with resveratrol in hypertensive patients. Sci. Rep..

[108] Fogacci F., Tocci G., Presta V., Fratter A., Borghi C., Cicero A.F.G. (2019). Effect of resveratrol on blood pressure: A systematic review and meta-analysis of randomized, controlled, clinical trials. Crit. Rev. Food Sci. Nutr..

[109] Voloshyna I., Hussaini S.M., Reiss A.B. (2012). Resveratrol in cholesterol metabolism and atherosclerosis. J. Med. Food.

[110] Huang K., Chen Z., Wang R., Ying H., Duan J., Zhang Y., Shi Q., Yang C., Yang L. (2025). Genetic targets related to aging for the treatment of coronary artery disease. BMC Med. Genom..

[111] Andriantsitohaina R., Auger C., Chataigneau T., Étienne-Selloum N., Li H., Martínez M.C., Schini-Kerth V.B., Laher I. (2012). Molecular mechanisms of the cardiovascular protective effects of polyphenols. Br. J. Nutr..

[112] Damay V.A., Ivan I. (2024). Resveratrol as an Anti-inflammatory Agent in Coronary Artery Disease: A Systematic Review, Meta-Analysis and Meta-Regression. Chin. J. Integr. Med..

[113] Gao Y., Fu R., Wang J., Yang X., Wen L., Feng J. (2018). Resveratrol mitigates the oxidative stress mediated by hypoxic-ischemic brain injury in neonatal rats via Nrf2/HO-1 pathway. Pharm. Biol..

[114] Liu J., He J., Huang Y., Hu Z. (2021). Resveratrol has an Overall Neuroprotective Role in Ischemic Stroke: A Meta-Analysis in Rodents. Front. Pharmacol..

[115] Fodor K., Tit D.M., Pasca B., Bustea C., Uivarosan D., Endres L., Iovan C., Abdel-Daim M.M., Bungau S. (2018). Long-Term Resveratrol Supplementation as a Secondary Prophylaxis for Stroke. Oxid. Med. Cell. Longev..

[116] Wang Q., Yu Q., Wu M. (2022). Antioxidant and neuroprotective actions of resveratrol in cerebrovascular diseases. Front. Pharmacol..

[117] Owjfard M., Rahimian Z., Karimi F., Borhani-Haghighi A., Mallahzadeh A. (2024). A comprehensive review on the neuroprotective potential of resveratrol in ischemic stroke. Heliyon.

[118] Fan S., Hu Y., You Y., Xue W., Chai R., Zhang X., Shou X., Shi J. (2022). Role of resveratrol in inhibiting pathological cardiac remodeling. Front. Pharmacol..

[119] Fang W.J., Wang C.J., He Y., Zhou Y.L., Peng X.D., Liu S.K. (2018). Resveratrol alleviates diabetic cardiomyopathy in rats by improving mitochondrial function through PGC-1α deacetylation. Acta Pharmacol. Sin..

[120] Gal R., Deres L., Horvath O., Eros K., Sandor B., Urban P., Soos S., Marton Z., Sumegi B., Toth K. (2020). Resveratrol Improves Heart Function by Moderating Inflammatory Processes in Patients with Systolic Heart Failure. Antioxidants.

[121] Riche D.M., McEwen C.L., Riche K.D., Sherman J.J., Wofford M.R., Deschamp D., Griswold M. (2013). Analysis of safety from a human clinical trial with pterostilbene. J. Toxicol..

[122] Brown V.A., Patel K.R., Viskaduraki M., Crowell J.A., Perloff M., Booth T.D., Vasilinin G., Sen A., Schinas A.M., Piccirilli G. (2010). Repeat dose study of the cancer chemopreventive agent resveratrol in healthy volunteers: Safety, pharmacokinetics, and effect on the insulin-like growth factor axis. Cancer Res..

[123] Plauth A., Geikowski A., Cichon S., Wowro S.J., Liedgens L., Rousseau M., Weidner C., Fuhr L., Kliem M., Jenkins G. (2016). Hormetic shifting of redox environment by pro-oxidative resveratrol protects cells against stress. Free Radic. Biol. Med..

[124] Detampel P., Beck M., Krähenbühl S., Huwyler J. (2012). Drug interaction potential of resveratrol. Drug Metab. Rev..

[125] Yang H., Wang Y., Wang Y., Tang K., Guo J., Li T. (2025). A review: Advances of resveratrol co-delivery biomaterials-based system in anti-tumor therapy. J. Mater Sci. Mater Med..

[126] Dikmetas D.N., Yenipazar H., Can Karaca A. (2024). Recent advances in encapsulation of resveratrol for enhanced delivery. Food Chem..

[127] Tain Y.L., Chang S.K.C., Liao J.X., Chen Y.W., Huang H.T., Li Y.L., Hou C.Y. (2021). Synthesis of Short-Chain-Fatty-Acid Resveratrol Esters and Their Antioxidant Properties. Antioxidants.

[128] Jacob S., Rao R., Gorain B., Boddu S.H.S., Nair A.B. (2025). Solid Lipid Nanoparticles and Nanostructured Lipid Carriers for Anticancer Phytochemical Delivery: Advances, Challenges, and Future Prospects. Pharmaceutics.

[129] Ramalingam P., Ko Y.T. (2015). Enhanced oral delivery of curcumin from N-trimethyl chitosan surface-modified solid lipid nanoparticles: Pharmacokinetic and brain distribution evaluations. Pharm. Res..

[130] Jøraholmen M.W., Škalko-Basnet N., Acharya G., Basnet P. (2015). Resveratrol-loaded liposomes for topical treatment of the vaginal inflammation and infections. Eur. J. Pharm. Sci..

[131] Zhang Y., Song H., Shang Z., Chen A., Huang D., Zhao H., Du H. (2014). Amino acid-PEGylated resveratrol and its influence on solubility and the controlled release behavior. Biol. Pharm. Bull..

[132] Ba S., Qiao M., Jia L., Zhang J., Zhao X., Hu H., Chen D. (2021). Construction of Hierarchical-Targeting pH-Sensitive Liposomes to Reverse Chemotherapeutic Resistance of Cancer Stem-like Cells. Pharmaceutics.

[133] Gregoriou Y., Gregoriou G., Yilmaz V., Kapnisis K., Prokopi M., Anayiotos A., Strati K., Dietis N., Constantinou A.I., Andreou C. (2021). Resveratrol loaded polymeric micelles for theranostic targeting of breast cancer cells. Nanotheranostics.

[134] Liu Q., Qin Y., Jiang B., Chen J., Zhang T. (2022). Development of self-assembled zein-fucoidan complex nanoparticles as a delivery system for resveratrol. Colloids Surf. B Biointerfaces.

[135] Sarma S., Agarwal S., Bhuyan P., Hazarika J., Ganguly M. (2022). Resveratrol-loaded chitosan-pectin core-shell nanoparticles as novel drug delivery vehicle for sustained release and improved antioxidant activities. R. Soc. Open Sci..

[136] Gu Y., Fei Z. (2022). Mesoporous Silica Nanoparticles Loaded with Resveratrol Are Used for Targeted Breast Cancer Therapy. J. Oncol..

[137] Machado T.F., Serra M.E.S., Murtinho D., Valente A.J.M., Naushad M. (2021). Covalent Organic Frameworks: Synthesis, Properties and Applications—An Overview. Polymers.

[138] Das P., Gupta M., Sahu A., Mukherjee T., Mohanty S., Nayak N., Kumari S., Singh R.P., Lariya D., Chopra M.P. (2025). Advances in Nanomedicine: Transforming Diagnostic Imaging with Novel Contrast Agents. Curr. Drug Metab..

[139] Li Z., Qiao W., Wang C., Wang H., Ma M., Han X., Tang J. (2020). DPPC-coated lipid nanoparticles as an inhalable carrier for accumulation of resveratrol in the pulmonary vasculature, a new strategy for pulmonary arterial hypertension treatment. Drug Deliv..

[140] Cui Q., Wang H. (2025). Resveratrol in Dermatological Therapy: A Critical Review of Mechanisms, Delivery Innovations, and Clinical Frontiers. Clin. Cosmet. Investig. Dermatol..

[141] Bavafa A., Sahab-Negah S., Forouzanfar F. (2025). Nose-to-Brain Targeting of Resveratrol Nanoformulations. Curr. Vasc. Pharmacol..

[142] Paczkowska-Walendowska M., Dvořák J., Rosiak N., Tykarska E., Szymańska E., Winnicka K., Ruchała M.A., Cielecka-Piontek J. (2021). Buccal Resveratrol Delivery System as a Potential New Concept for the Periodontitis Treatment. Pharmaceutics.

[143] Amen T., Guihur A., Zelent C., Ursache R., Wilting J., Kaganovich D. (2021). Resveratrol and related stilbene derivatives induce stress granules with distinct clearance kinetics. Mol. Biol. Cell..

[144] Ruparelia K.C., Zeka K., Beresford K.J.M., Wilsher N.E., Potter G.A., Androutsopoulos V.P., Brucoli F., Arroo R.R.J. (2024). CYP1-Activation and Anticancer Properties of Synthetic Methoxylated Resveratrol Analogues. Molecules.

[145] Liang L., Liu X., Wang Q., Cheng S., Zhang S., Zhang M. (2013). Pharmacokinetics, tissue distribution and excretion study of resveratrol and its prodrug 3,5,4′-tri-O-acetylresveratrol in rats. Phytomedicine.

[146] Chen H., Huang L., Li X., Du J., Wang Z., Chen L., Zheng P., Nie C., Yin M., Zhu W. (2026). Interfacial Engineering Strategies in Bio-heterojunctions for Antibacterial Therapeutics and Biomedical Applications. Acta Biomater..

[147] Intagliata S., Modica M.N., Santagati L.M., Montenegro L. (2019). Strategies to Improve Resveratrol Systemic and Topical Bioavailability: An Update. Antioxidant.

[148] Ruan B.F., Lu X.Q., Song J., Zhu H.L. (2012). Derivatives of resveratrol: Potential agents in prevention and treatment of cardiovascular disease. Curr. Med. Chem..

[149] Nawaz W., Zhou Z., Deng S., Ma X., Ma X., Li C., Shu X. (2017). Therapeutic Versatility of Resveratrol Derivatives. Nutrients.

[150] Hsu C.N., Lin Y.J., Hou C.Y., Chen Y.W., Tain Y.L. (2025). Early-Life Prevention of Cardiovascular-Kidney-Metabolic Syndrome: The DOHaD Perspective on Resveratrol and Short-Chain Fatty Acids. Antioxidants.

[151] Magliocca G., Mone P., Di Iorio B.R., Heidland A., Marzocco S. (2022). Short-Chain Fatty Acids in Chronic Kidney Disease: Focus on Inflammation and Oxidative Stress Regulation. Int. J. Mol. Sci..

[152] González-Bosch C., Boorman E., Zunszain P.A., Mann G.E. (2021). Short-chain fatty acids as modulators of redox signaling in health and disease. Redox Biol..

[153] Neises B., Steglich W. (1978). Simple Method for the Esterification of Carboxylic Acids. Angew. Chem. Int. Ed..

[154] Tain Y.L., Jheng L.C., Chang S.K.C., Chen Y.W., Huang L.T., Liao J.X., Hou C.Y. (2020). Synthesis and Characterization of Novel Resveratrol Butyrate Esters That Have the Ability to Prevent Fat Accumulation in a Liver Cell Culture Model. Molecules.

[155] Kelishadi R., Poursafa P. (2014). A review on the genetic, environmental, and lifestyle aspects of the early-life origins of cardiovascular disease. Curr. Probl. Pediatr. Adolesc. Health Care.

[156] Tain Y.L., Hsu C.N. (2017). Developmental Origins of Chronic Kidney Disease: Should We Focus on Early Life?. Int. J. Mol. Sci..

[157] McMillen I.C., Robinson J.S. (2005). Developmental origins of the metabolic syndrome: Prediction, plasticity, and programming. Physiol. Rev..

[158] Tain Y.L., Lee W.C., Wu K.L.H., Leu S., Chan J.Y.H. (2018). Resveratrol Prevents the Development of Hypertension Programmed by Maternal Plus Post-Weaning High-Fructose Consumption through Modulation of Oxidative Stress, Nutrient-Sensing Signals, and Gut Microbiota. Mol. Nutr. Food Res..

[159] Hsu C.N., Hou C.Y., Chang-Chien G.P., Lin S., Yang H.W., Tain Y.L. (2020). Perinatal Resveratrol Therapy Prevents Hypertension Programmed by Maternal Chronic Kidney Disease in Adult Male Offspring: Implications of the Gut Microbiome and Their Metabolites. Biomedicines.

[160] Hsu C.N., Hou C.Y., Chang-Chien G.P., Lin S., Chan J.Y.H., Lee C.T., Tain Y.L. (2021). Maternal resveratrol therapy protected adult rat offspring against hypertension programmed by combined exposures to asymmetric dimethylarginine and trimethylamine-N-oxide. J. Nutr. Biochem..

[161] Hsu C.N., Hung C.H., Hou C.Y., Chang C.I., Tain Y.L. (2021). Perinatal Resveratrol Therapy to Dioxin-Exposed Dams Prevents the Programming of Hypertension in Adult Rat Offspring. Antioxidants.

[162] Chen H.E., Lin Y.J., Lin I.C., Yu H.R., Sheen J.M., Tsai C.C., Huang L.T., Tain Y.L. (2019). Resveratrol prevents combined prenatal N^G^-nitro-L-arginine-methyl ester (L-NAME) treatment plus postnatal high-fat diet induced programmed hypertension in adult rat offspring: Interplay between nutrient-sensing signals, oxidative stress and gut microbiota. J. Nutr. Biochem..

[163] Hsu C.N., Lin Y.J., Lu P.C., Tain Y.L. (2018). Maternal resveratrol therapy protects male rat offspring against programmed hypertension induced by TCDD and dexamethasone exposures: Is it relevant to aryl hydrocarbon receptor?. Int. J. Mol. Sci..

[164] Hsu C.N., Lin Y.J., Tain Y.L. (2019). Maternal Exposure to Bisphenol A Combined with High-Fat Diet-Induced Programmed Hypertension in Adult Male Rat Offspring: Effects of Resveratrol. Int. J. Mol. Sci..

[165] Tain Y.L., Lin Y.J., Sheen J.M., Lin I.C., Yu H.R., Huang L.T., Hsu C.N. (2017). Resveratrol prevents the combined maternal plus post weaning high-fat-diets-induced hypertension in male offspring. J. Nutr. Biochem..

[166] Care A.S., Sung M.M., Panahi S., Gragasin F.S., Dyck J.R., Davidge S.T., Bourque S.L. (2016). Perinatal Resveratrol Supplementation to Spontaneously Hypertensive Rat Dams Mitigates the Development of Hypertension in Adult Offspring. Hypertension.

[167] Sheen J.M., Yu H.R., Tain Y.L., Tsai W.L., Tiao M.M., Lin I.C., Tsai C.C., Lin Y.J., Huang L.T. (2018). Combined maternal and postnatal high-fat diet leads to metabolic syndrome and is effectively reversed by resveratrol: A multiple-organ study. Sci. Rep..

[168] Ros P., Díaz F., Freire-Regatillo A., Argente-Arizón P., Barrios V., Argente J., Chowen J.A. (2018). Resveratrol Intake during Pregnancy and Lactation Modulates the Early Metabolic Effects of Maternal Nutrition Differently in Male and Female Offspring. Endocrinology.

[169] Zou T., Chen D., Yang Q., Wang B., Zhu M.J., Nathanielsz P.W., Du M. (2017). Resveratrol supplementation of high-fat diet-fed pregnant mice promotes brown and beige adipocyte development and prevents obesity in male offspring. J. Physiol..

[170] Vega C.C., Reyes-Castro L.A., Rodríguez-González G.L., Bautista C.J., Vázquez-Martínez M., Larrea F., Chamorro-Cevallos G.A., Nathanielsz P.W., Zambrano E. (2016). Resveratrol partially prevents oxidative stress and metabolic dysfunction in pregnant rats fed a low protein diet and their offspring. J. Physiol..

[171] Pound L.D., Comstock S.M., Grove K.L. (2014). Consumption of a Western-style diet during pregnancy impairs offspring islet vascularization in a Japanese macaque model. Am. J. Physiol. Endocrinol. Metab..

[172] Roberts V.H., Pound L.D., Thorn S.R., Gillingham M.B., Thornburg K.L., Friedman J.E., Frias A.E., Grove K.L. (2014). Beneficial and cautionary outcomes of resveratrol supplementation in pregnant nonhuman primates. FASEB J..

[173] Diaz-Gerevini G.T., Repossi G., Dain A., Tarres M.C., Das U.N., Eynard A.R. (2016). Beneficial action of resveratrol: How and why?. Nutrition.

[174] Tain Y.L., Hsu C.N. (2018). AMP-Activated protein kinase as a reprogramming strategy for hypertension and kidney disease of developmental origin. Int. J. Mol. Sci..

[175] Stevens E.A., Mezrich J.D., Bradfield C.A. (2009). The aryl hydrocarbon receptor: A perspective on potential roles in the immune system. Immunology.

[176] Sallée M., Dou L., Cerini C., Poitevin S., Brunet P., Burtey S. (2014). The aryl hydrocarbon receptor-activating effect of uremic toxins from tryptophan metabolism: A new concept to understand cardiovascular complications of chronic kidney disease. Toxins.

[177] Frombaum M., Therond P., Djelidi R., Beaudeux J.L., Bonnefont-Rousselot D., Borderie D. (2011). Piceatannol is more effective than resveratrol in restoring endothelial cell dimethylarginine dimethylaminohydrolase expression and activity after high-glucose oxidative stress. Free Radic. Res..

[178] Liu X., Xiong H., Lu M., Liu B., Hu C., Liu P. (2024). Trans-3, 5, 4'-trimethoxystilbene restrains non-small-cell lung carcinoma progression via suppressing M2 polarization through inhibition of m6A modified circPACRGL-mediated Hippo signaling. Phytomedicine.

[179] Shih M.K., Tain Y.L., Chen Y.W., Hsu W.H., Yeh Y.T., Chang S.K.C., Liao J.X., Hou C.Y. (2021). Resveratrol Butyrate Esters Inhibit Obesity Caused by Perinatal Exposure to Bisphenol A in Female Offspring Rats. Molecules.

[180] Liao J.X., Chen Y.W., Shih M.K., Tain Y.L., Yeh Y.T., Chiu M.H., Chang S.K.C., Hou C.Y. (2021). Resveratrol Butyrate Esters Inhibit BPA-Induced Liver Damage in Male Offspring Rats by Modulating Antioxidant Capacity and Gut Microbiota. Int. J. Mol. Sci..

[181] Tain Y.L., Hou C.Y., Chang-Chien G.P., Lin S., Hsu C.N. (2023). Resveratrol Butyrate Ester Supplementation Blunts the Development of Offspring Hypertension in a Maternal Di-2-ethylhexyl Phthalate Exposure Rat Model. Nutrients.

[182] Tain Y.L., Hou C.Y., Tzeng H.T., Lin S.F., Chang-Chien G.P., Lee W.C., Wu K.L.H., Yu H.R., Chan J.Y.H., Hsu C.N. (2024). Effect of Purified Resveratrol Butyrate Ester Monomers against Hypertension after Maternal High-Fructose Intake in Adult Offspring. Nutrients.

[183] Pasciu V., Posadino A.M., Cossu A., Sanna B., Tadolini B., Gaspa L., Marchisio A., Dessole S., Capobianco G., Pintus G. (2010). Akt downregulation by flavin oxidase—Induced ROS generation mediates dose-dependent endothelial cell damage elicited by natural antioxidants. Toxicol. Sci..

[184] Posadino A.M., Cossu A., Giordo R., Zinellu A., Sotgia S., Vardeu A., Hoa P.T., Carru C., Pintus G. (2015). Resveratrol alters human endothelial cells redox state and causes mitochondrial-dependent cell death. Food Chem. Toxicol..

[185] Salehi B., Mishra A.P., Nigam M., Sener B., Kilic M., Sharifi-Rad M., Fokou P.V.T., Martins N., Sharifi-Rad J. (2018). Resveratrol: A double-edged sword in health benefits. Biomedicines.

[186] Savchuk I., Morvan M.L., Søeborg T., Antignac J.P., Gemzell-Danielsson K., Le Bizec B., Söder O., Svechnikov K. (2017). Resveratrol inhibits steroidogenesis in human fetal adrenocortical cells at the end of first trimester. Mol. Nutr. Food Res..

[187] Brown K., Theofanous D., Britton R.G., Aburido G., Pepper C., Sri Undru S., Howells L. (2024). Resveratrol for the Management of Human Health: How Far Have We Come? A Systematic Review of Resveratrol Clinical Trials to Highlight Gaps and Opportunities. Int. J. Mol. Sci..

[188] Darby J.R.T., Mohd Dollah M.H.B., Regnault T.R.H., Williams M.T., Morrison J.L. (2019). Systematic review: Impact of resveratrol exposure during pregnancy on maternal and fetal outcomes in animal models of human pregnancy complications-Are we ready for the clinic?. Pharmacol. Res..

[189] Ramli I., Posadino A.M., Giordo R., Fenu G., Fardoun M., Iratni R., Eid A.H., Zayed H., Pintus G. (2023). Effect of Resveratrol on Pregnancy, Prenatal Complications and Pregnancy-Associated Structure Alterations. Antioxidants.

[190] Smoliga J.M., Vang O., Baur J.A. (2012). Challenges of translating basic research into therapeutics: Resveratrol as an example. J. Gerontol. A Biol. Sci. Med. Sci..

[191] Liu Y., Ma W., Zhang P., He S., Huang D. (2015). Effect of resveratrol on blood pressure: A meta-analysis of randomized controlled trials. Clin. Nutr..

[192] Hausenblas H.A., Schoulda J.A., Smoliga J.M. (2015). Resveratrol treatment as an adjunct to pharmacological management in type 2 diabetes mellitus--systematic review and meta-analysis. Mol. Nutr. Food Res..

[193] Zhang L.X., Li C.X., Kakar M.U., Khan M.S., Wu P.F., Amir R.M., Dai D.F., Naveed M., Li Q.Y., Saeed M. (2021). Resveratrol (RV): A pharmacological review and call for further research. Biomed. Pharmacother..

[194] Zeng P., Huang H., Li D. (2024). Combining bioinformatics, network pharmacology, and artificial intelligence to predict the mechanism of resveratrol in the treatment of rheumatoid arthritis. Heliyon.

[195] Gong W., Sun P., Li X., Wang X., Zhang X., Cui H., Yang J. (2024). Investigating the Molecular Mechanisms of Resveratrol in Treating Cardiometabolic Multimorbidity: A Network Pharmacology and Bioinformatics Approach with Molecular Docking Validation. Nutrients.

[196] Zhang Y., Cheng J., Jian W. (2025). Unlocking Resveratrol’s Potential: Targeting Ferroptosis in Atherosclerosis Through MAPK1. Food Sci. Nutr..

[197] Liu Q., Wang T., Nian B., Ma F., Zhao S., Vásquez A.F., Guo L., Ding C., Davari M.D. (2026). Decoding polyphenol-protein interactions with deep learning: From molecular mechanisms to food applications. Biotechnol. Adv..

[198] OMIM. https://www.omim.org/.

[199] GeneCards. https://www.genecards.org/.

[200] DisGeNET. https://www.disgenet.org/.

[201] TCMSP. https://www.tcmsp-e.com/load_intro.php?id=43.

[202] DrugBank. https://go.drugbank.com/.

[203] STRING. https://cn.string-db.org/.

[204] DAVID Online Platform. https://davidbioinformatics.nih.gov/.

[205] Barrett T., Wilhite S.E., Ledoux P., Evangelista C., Kim I.F., Tomashevsky M., Marshall K.A., Phillippy K.H., Sherman P.M., Holko M. (2013). NCBI GEO: Archive for functional genomics data sets-update. Nucleic Acids Res..

[206] David A., Islam S., Tankhilevich E., Sternberg M.J.E. (2022). The AlphaFold Database of Protein Structures: A Biologist’s Guide. J. Mol. Biol..

[207] Ros H., Chan N., Cook M.T., Shorthouse D. (2026). Artificial intelligence and machine learning guided optimization in drug delivery. Adv. Drug Deliv. Rev..

[208] Tubbs A., Vazquez E.A. (2025). Digital twins in increasing diversity in clinical trials: A systematic review. J. Biomed. Inform..

[209] Mlakić M., Odak I., Barić D., Talić S., Šagud I., Štefanić Z., Molčanov K., Lasić Z., Kovačević B., Škorić I. (2024). New resveratrol analogs as improved biologically active structures: Design, synthesis and computational modeling. Bioorg. Chem..

